# Combination of Copper Metallodendrimers with Conventional Antitumor Drugs to Combat Cancer in In Vitro Models

**DOI:** 10.3390/ijms24044076

**Published:** 2023-02-17

**Authors:** Marcin Hołota, Sylwia Michlewska, Sandra Garcia-Gallego, Natalia Sanz del Olmo, Paula Ortega, Maria Bryszewska, Francisco Javier de la Mata, Maksim Ionov

**Affiliations:** 1 Department of General Biophysics, Faculty of Biology & Environmental Protection, University of Lodz, Pomorska 141/143, 90-236 Lodz, Poland; 2 Laboratory of Microscopic Imaging & Specialized Biological Techniques, Faculty of Biology & Environmental Protection, University of Lodz, Banacha12/16, 90-237 Lodz, Poland; 3 Department of Organic and Inorganic Chemistry, Research Institute in Chemistry “Andrés M. del Río” (IQAR), Universidad de Alcalá, 28805 Madrid, Spain; 4 Networking Research Center on Bioengineering, Biomaterials and Nanomedicine (CIBER-BBN), 28029 Madrid, Spain; 5 Institute Ramón y Cajal for Health Research (IRYCIS), 28034 Madrid, Spain

**Keywords:** dendrimers, copper, anticancer drug, drug delivery, apoptosis, necrosis

## Abstract

Copper carbosilane metallodendrimers containing chloride ligands and nitrate ligands were mixed with commercially available conventional anticancer drugs, doxorubicin, methotrexate and 5-fluorouracil, for a possible therapeutic system. To verify the hypothesis that copper metallodendrimers can form conjugates with anticancer drugs, their complexes were biophysically characterized using zeta potential and zeta size methods. Next, to confirm the existence of a synergetic effect of dendrimers and drugs, in vitro studies were performed. The combination therapy has been applied in two cancer cell lines: MCF-7 (human breast cancer cell line) and HepG2 (human liver carcinoma cell line). The doxorubicin (DOX), methotrexate (MTX) and 5-fluorouracil (5-FU) were more effective against cancer cells when conjugated with copper metallodendrimers. Such combination significantly decreased cancer cell viability when compared to noncomplexed drugs or dendrimers. The incubation of cells with drug/dendrimer complexes resulted in the increase of the reactive oxygen species (ROS) levels and the depolarization of mitochondrial membranes. Copper ions present in the dendrimer structures enhanced the anticancer properties of the whole nanosystem and improved drug effects, inducing both the apoptosis and necrosis of MCF-7 (human breast cancer cell line) and HepG2 (human liver carcinoma cell line) cancer cells.

## 1. Introduction

Cancer is a serious global disease and is the second most common reason of death in the world [1,2]. The most commonly used methods of treatment for cancer are chemotherapy, radiation therapy and surgical excision of affected tissues [3]. However, commonly practiced therapies are not always effective, and many side effects connected with the treatment of cancers can negatively affect the quality of the patient’s life [2].

Currently, many studies are focusing on the development of anticancer drug carriers to improve their bioavailability and increase selectivity, thereby reducing side effects [4]. Using nanoparticle-based delivery systems can result in the increase of drug concentration in tumor tissue and a reduction of toxicity in healthy tissues. Presently, non-viral drug delivery involves the use of various types of nanoparticles, including polymeric nanoparticles, micelles, liposomes, nanotubes or dendrimers [5].

Dendrimers, due to their unique properties such as specific branching structures, monodispersity, thermal/chemical stability and hydrophobic/hydrophilic natures, have promise. Cationic dendrimers could be good candidates for use in chemotherapeutical transport systems [6].

Doxorubicin (DOX), methotrexate (MTX) and 5-fluorouracil (5-FU) are drugs that are commonly used in cancer chemotherapy [7,8,9]. Doxorubicin, by intercalating into DNA (deoxyribonucleic acid), inhibits the replication and transcription of cancer cells [10,11]. Although doxorubicin is one of the most effective anticancer drugs, its use is limited due to side effects such as cardiotoxicity, which can cause cardiomyopathies with fatal consequences [4]. The mechanism of methotrexate is similar and causes replication and transcription arrest, blocking cells developing in the G1 phase of the cell cycle [12]. Again, even though methotrexate is one of the most effective chemotherapeutics, it has dangerous side effects. The consequence of methotrexate application is myelosuppression, hepatotoxicity and pulmonary fibrosis [13]. 5-fluorouracil causes the damage of DNA and RNA (ribonucleic acid) of cancer cells [14] and is also cytotoxic against normal cells and tissues and can cause life-threatening cardiotoxicity [15].

Drugs used in classical chemotherapy do not distribute to specific parts of the body; therefore, they affect all cells, not just cancer cells. Consequently, there is a limitation of the possible doses for cancer treatment [5]. Additionally, many types of cancer cells are drug-resistant due to quick removal of the therapeutic material from the cytoplasm [16]. Delayed release of anticancer drugs from the complex with nanomaterials would be a desired effect.

A combined therapy of drugs to reduce side effects provides good results since, in combination, lower doses of the drugs are necessary to be effective. Moreover, the use of controlled release systems such as nanomaterials to increase the bioavailability of antimetastatic drugs seems to be promising.

Dendrimers, well-defined macromolecular structures, have proven to be good transfection vehicles and have been studied in combination therapy. Dendrimers can also undergo continuous modifications directed to increase their efficiency in cancer therapy. According to the literature, hydrophilic nanoparticle surface chemistry has been characterized as less toxic and has exhibited better biocompatibility than the cationic surface of nanoparticles for medical applications [17]. However, Shcharbin’s team displayed that only high generation of dendrimers with unmodified surface in high doses has some toxicity in vivo. The modifications of nanoparticle surface decrease their toxicity and determine the desired location of multifunctional dendrimer-based conjugates [18]. Additionally, the effects depend on the changing surface properties that influence the uptake of nanoparticles and their conjugates with drugs [17,19].

These modifications include the introduction of metal atoms that exhibit anticancer properties, for example, platinum, gold, silver, ruthenium or copper [6,20], into the dendrimer structure to improve their biomedical applications. In particular, carbosilane copper metallodendrimers bearing iminopyridine moieties have demonstrated outstanding anticancer activity in both solid tumors and myeloid cell lines [6,21,22,23,24].

It is known that copper is one of the most important micronutrients that takes part in biological oxidation-reduction (redox) reactions employed by critical enzymes, including cytochrome c oxidase (COX), NADH dehydrogenase-2 (ND2) or Cu/Zn–superoxide dismutase (SOD1). Additionally, it possesses redox activity that leads to the production of reactive oxygen species (ROS), which is connected with homeostatic regulation of copper within the body [25]. Invalid copper levels have been repeatedly reported about in cancer tissues [26,27,28].

Subtle changes, such as the metal counterion or the presence of ligands on the iminopyridine ring, can modulate antitumor activity, probably through different ROS-production pathways. This exemplifies the importance of adequately designing the metallodendrimer.

Several examples of the use of metallodendrimers in combination therapy are, for example, the combination of a third-generation copper (II) metallophosphate dendrimer in combination with DOX, cisplatin, paclitaxel and MG132, which showed stronger inhibition of HL60 cell proliferation compared to drugs and dendrimer use alone. The combination of ruthenium(II) carbosilane metallodendrimers showed synergism when used in combination therapy with MTX, DOX or 5-Fu against leukemia [16].

In the present study, copper carbosilane metallodendrimers were chosen for their conjugation with the previously mentioned anticancer drugs. Some preclinical and clinical studies show the positive effects of copper in cancer therapy [25] due to copper limiting many aspects such as growth, angiogenesis and metastasis of cancer progression [29]. Additionally, copper increases the level of reactive oxygen species (ROS) in cancer cells, the consequence of ROS generation being damage to DNA [23]. It is presumed that the ability to produce ROS underlies the copper toxic effects toward cancer cells that is not observed in normal cells. This could result in higher sensitivity of cancer cells for changes in ROS levels than normal cells [25].

This study presents the first results from experiments analyzing the effects of copper carbosilane metallodendrimers containing chloride Gn-[NCPh(*o*-N)CuCl_2_·H_2_O]_m_ (n = 0, m = 1 CCD-Cl-0; n = 1, m = 4 CCD-Cl-1; n = 2, m = 8 CCD-Cl-2) and nitrate ligands Gn-[NCPh(*o*-N)Cu(ONO_2_)_2_·H_2_O]m (n = 0, m = 1 CCD-NO-0; n = 1, m = 4 CCD-NO-1; n = 2, m = 8 CCD-NO-2) conjugated with conventional anticancer drugs against cancer cells. It was shown that studied dendrimers are more toxic towards cancer than normal cells and were able to improve the anticancer effect of traditional drugs.

## 2. Results

### 2.1. Zeta Potential and Size

**Figure 1:**
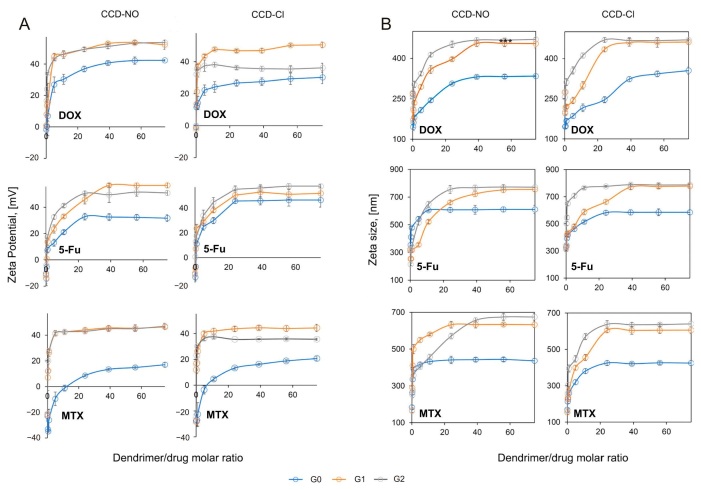
Zeta potential (**A**) and zeta average size (**B**) of DOX—top panels; 5-FU—middle panels; and MTX—bottom panels, at the presence of increasing concentrations of copper dendrimers. Drugs concentration 10 µmol/L. The measurements were performed using sodium phosphate buffer 10 mmol/L, pH 7.4. Results are mean ± standard deviation (SD), n = 3.

It is known that the surface charge, size and binding parameters of nanovectors are significant when evaluating them for drug carriers. Based on the previously published results of carbosilane metallodendrimer interaction with drugs [16], it was hypothesized that copper metallodendrimers can form complexes with DOX, MTX and 5-Fu. To prove this hypothesis, the size and surface charge of naked drugs were checked and then complexed with dendrimers. To determine the mentioned parameters, the zeta technique was applied. Obtained results indicate that both zeta size and zeta potential values were increased when dendrimers were added to the drug solution in all tested formulations. The addition of the CCD-NO of first and second generations (CCD-NO-1 and CCD-NO-2) to the drug suspension increased the zeta potential of the formed nanosystem up to 50 mV, while the presence of CCD-NO of generation “0” did not exceed 40 mV, giving the lowest values of size and zeta potential of all of them (see Figure 1).

The highest zeta potential was registered for the formulation DOX/CCD-NO-1 (Figure 1A). Similar results were obtained for the complexes formed by CCD-Cl dendrimers with drugs. While CCD-NO-1,2 and CCD-Cl-1,2 dendrimers were added to the MTX solution, the zeta potential values were near 40 mV. The dendrimers of generation “0” again showed the lowest values not exceeding 20 mV. CCD-Cl dendrimers exhibit a less cationic character with more lipophilic properties than CCD-NO, due to the stronger Cu-Cl bond. This behavior will impact the stability of the complexes formed with each of the drugs, which intrinsically exhibit different properties. For example, doxorubicin and methotrexate show pending ionizable groups and nicely interact with CCD-NO with no relevant differences between G1 and G2. However, the increase in lipophilicity from CCD-Cl-1 to CCD-Cl-2 is counterproductive in the interaction with DOX and MTX (but slightly favorable with 5-FU).

The size of nanocomplexes formed with CCD-NO and CCD-Cl dendrimers of generations one and two with DOX, 5-FU and MTX were near 450 nm, 800 nm and 600 nm, respectively. The compounds of generation “0” formed smaller complexes with the size being about 300 nm for DOX, 600 nm for 5-FU and 400 nm for MTX, as expected (Figure 1B).

### 2.2. Cytotoxicity

**Figure 2:**
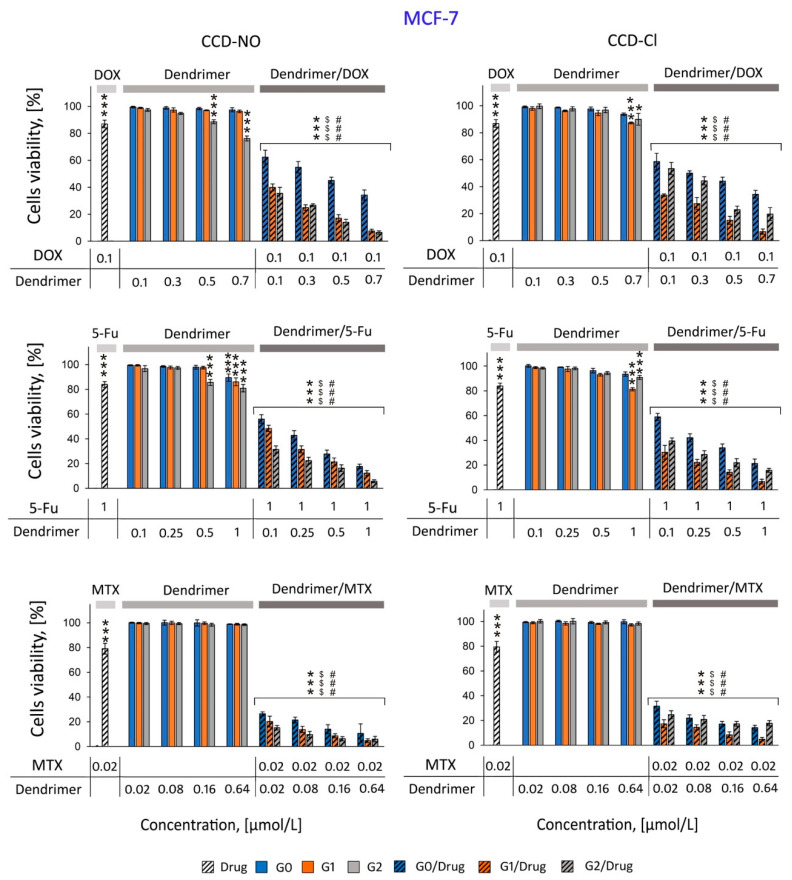
Cytotoxicity profiles of anticancer drugs, copper dendrimers and their (dendrimer/drug) complexes towards MCF7 cells. MTT assay, incubation time 72 h in phosphate saline buffer 10 mmol/L, pH 7.4. Results are means ± SD, from a min. 3 independent experiments. Statistically significant differences vs. control: * *p* <0.05, *** *p* < 0.001, vs. free drug $$$ *p* < 0.001, vs. free dendrimer ### *p* < 0.001.

**Figure 3:**
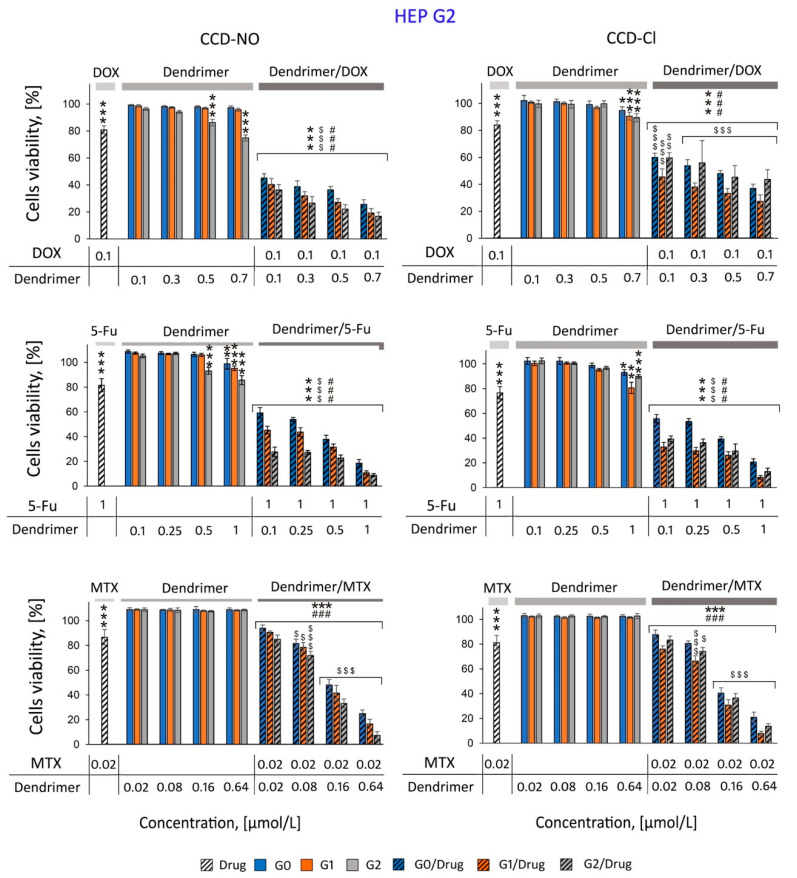
Cytotoxicity profiles of anticancer drugs, copper dendrimers and their (dendrimer/drug) complexes towards HEP G2 cells. MTT assay, incubation time 72 h in phosphate saline buffer 10 mmol/L, pH 7.4. Results are means ± SD, from a min. 3 independent experiments. Statistically significant differences vs. control: * *p* <0.05, ** *p* < 0.01, *** *p* < 0.001, vs. free drug $ *p* <0.05, $$$ *p* < 0.001, vs. free dendrimer ### *p* < 0.001.

Since the main aim of this study was to analyze the synergetic effect of complexes together with traditional anticancer drugs with copper metallodendrimers and additionally show the suitability of using CCDs as drug carriers, the experiments on cell viability with the presence of both components were performed in two different lines: MCF-7 (Figure 2) and HepG2 cells (Figure 3).

The MCF7 and HepG2 cells were incubated for 72 h with drugs, dendrimers or drug/dendrimer complexes. The amount of drugs established on the base of drug effective concentrations (80% of viability for considered cells) was as follows: DOX 0.1 µmol/L, 5-FU 1µmol/L and MTX 0.02 nmol/L.

The results presented in Figure 2 and Figure 3 show significant synergistic effect of copper metallodendrimers complexed with drugs. All tested concentrations of the dendritic systems and individual drugs were shown to be of low cytotoxicity in both cell lines, MCF7 (Figure 3) and HepG2 (Figure 3), while their combination (dendrimer/drug) in different concentrations significantly reduced cell viability. The synergistic effect is more pronounced in the combination with doxorubicin and 5-fluorouracil, where it is possible to observe reductions of 40 to 80% with respect to the control, as the concentration of the dendritic system in the formulation is increased.

It is worth highlighting that among CCD-NO dendrimers, antitumor activity increases with the dendrimer generation, G2 being the better carrier. However, among CCD-Cl dendrimers, it is the first-generation metallodendrimer which is the most efficient. This behavior highlights the impact of the metal counterion, with chloride generating a more hydrophobic environment which improves the interaction with the drugs, even at lower generations.

The complexes formed by all drugs with CCD-NO and CCD-Cl dendrimers had similar effects; however, the cytotoxicity of CCD-NO formulations was slightly higher, maybe due to the major solubility of dendrimers in aqueous medium when the copper ligand is nitrate. The most cytotoxic CCD-NO-1/MTX formulation in the highest applied concentration reduced the number of living cells by 95.25% vs. the control. The other tendency was observed for 5-FU/dendrimer nanocomplexes. While CCD-NO complexes showed generation-dependent cytotoxic activity, among the CCD-Cl formulations the most effective was the system formed with the first generation of dendrimers. CCD-NO-2/5-FU reduced the number of living cells by 94.37% and 91.92% for MCF7 and HEPG2 cells, respectively, and the CCD-Cl-1/5-FU decreased the viability of cells by 93.38% for MCF7 and 92.02% for HEPG2 cells. Similarly, while CCD-NO/DOX complexes decreased the viability of both cell lines in a generation-dependent manner, among the CCD-Cl/DOX systems the most cytotoxic was CCD-Cl-1/DOX. The number of living cells decreased by about 90% for MCF7 and by about 70% for HEPG2 cells.

### 2.3. Generation of Intracellular Reactive Oxygen Species (ROS)

**Figure 4:**
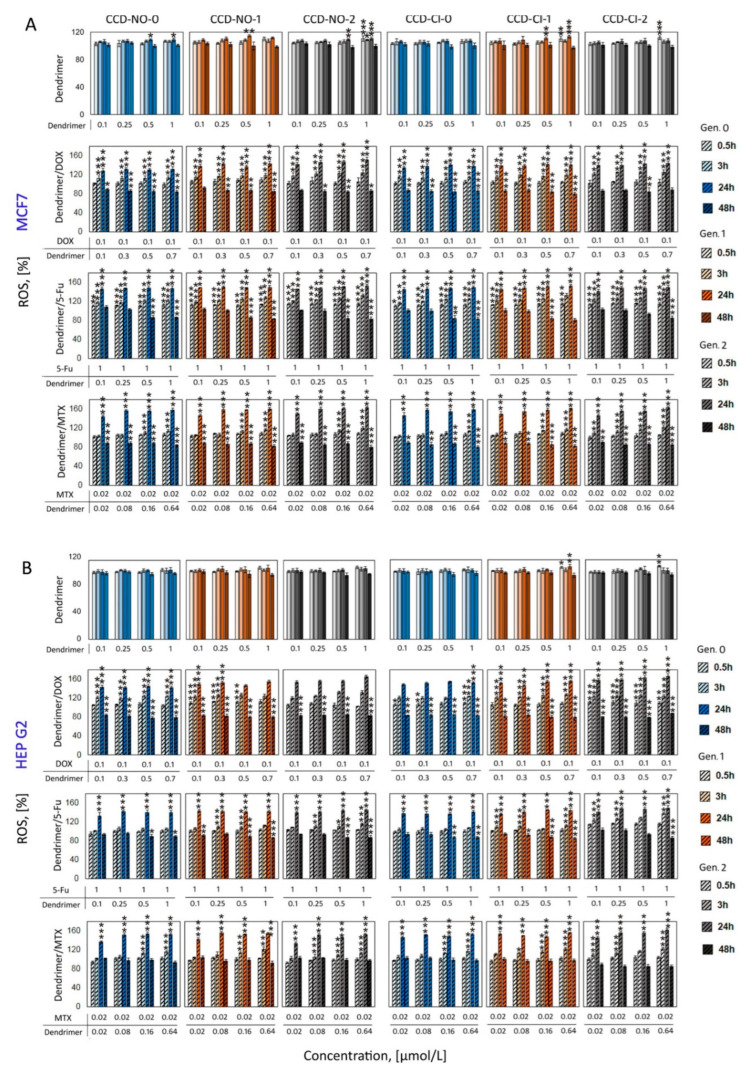
Time-dependent ROS production in MCF7 (**A**) or HEP G2 (**B**) cells induced by copper dendrimers or their complexes with anticancer drugs DOX, 5-Fu and MTX. Fluorescent probe H_2_DCFDA 2 µmol/L, incubation time 0.5 h, 3 h, 24 h, 48 h, phosphate-buffered saline 10 mmol/L, pH 7.4. Results are means ± SD, from a min. 3 independent experiments. Statistically significant differences vs. control: * *p* < 0.05, ** *p* < 0.01, *** *p* < 0.001. Control (untreated cells) is 100% ROS production.

Carbosilane metallodendrimers have cytotoxic responses towards cancer cells [6,30,31,32] that are consistent with our present studies on cell viability described above. To understand the toxicity mechanisms of the dendrimers and their drug complexes better, several additional analyses were performed. One of the studies carried out was the evaluation of an in vitro generation of intracellular reactive oxygen species (ROS) where MCF 7 and HEP G2 cancer cells are exposed to copper metallodendrimers and combination dendrimer/drugs. An increased production of intracellular ROS level due to in vitro exposure of MCF 7 and HEP G2 cancer cells to copper dendrimers and analyzed dendrimer/drug complexes was evaluated. The levels of ROS were detected at 0.5, 3, 24 and 48 h incubation of cells with studied samples. Neither of the used dendrimer concentrations significantly increased the level of ROS in both tested cell lines during applied incubation time (Figure 4).

The figure of ROS level changes involved by naked drugs is present in the Supplementary File (Figure S1A). When dendrimers were complexed with drugs and added to the cell suspension, the data showed that although ROS production initially increased in an approximately linear fashion at the beginning, at 24 h of incubation there is maximum ROS production, with decreases after 48 h of incubation. Interestingly, the application of higher doses only slightly influenced the ROS level. The results are similar in both MCF7 and HepG2 cells, the highest increase in the generation of ROS being observed after 24 h of incubation, and in selected cases a massive increase in the ROS level has been observed: 160% vs. control (100%).

### 2.4. Mitochondrial Membrane Potential

**Figure 5:**
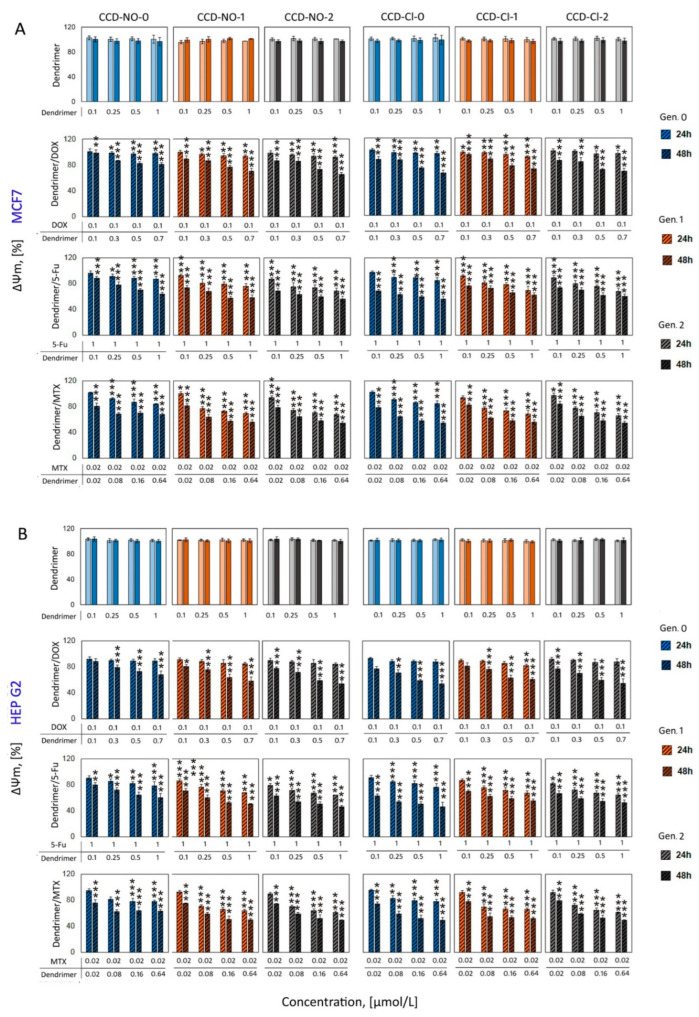
Changes in the mitochondrial membrane potential (ΔΨ_m_) in MCF7 (**A**) and HEP G2 (**B**) cells after exposure to copper dendrimers or their complexes with anticancer drugs DOX, 5-Fu and MTX, measured by JC-1 fluorescent probe in PBS10 mmol/L, pH 7.4. Results are means ± SD, from a min. 3 independent experiments. Statistically significant differences vs. control: * *p* < 0.05, ** *p* < 0.01, *** *p* < 0.001.

After the analysis of the reactive oxygen species formation, alterations in mitochondrial membrane potential were studied. Carbosilane copper metallodendrimers have been reported to selectively alter the mitochondrial membrane potential in tumor cells [24]. At 10 µmol/L, the metallodendrimers produced a pro-oxidative effect at 24–48 h in U937 cells, which collapsed at 48 h and continued decreasing until 72 h. This effect was different among nitrate and chloride dendrimers and also depended on the generation. In PBMC, a similar effect was observed, but from 48 h a recovery was observed. In this study, a JC-1 fluorescent probe was used to determine the mitochondrial membrane potential changes after 24 and 48 h incubation of cells with copper dendrimers, drugs or dendrimer/drug complexes. Results show that changes in mitochondrial membrane potential were not significant in cells treated with noncomplexed dendrimers at low concentrations (0.02–0.64 µmol/L) (Figure 5) or naked drugs (Figure S1B), regardless of the concentrations tested or the applied incubation time.

The 24 h incubation of cells with dendrimers/drug complexes just slightly decreased the mitochondrial potential of cells of both used cell lines. After 48 h of incubation, this effect was more pronounced for all formulations studied (Figure 5), showing that this effect depends on the dendrimer concentration but not on the dendrimer generation. The biggest drop in Ψ_m_ was observed for second-generation dendrimer CCD-2/MTX formulation, independent of the ligand nature (NO or Cl). The mitochondrial potential decreased by 40% for MCF7 and 35% for HEPG2 cells vs. control untreated cells. The results showed that the highest concentrations of complexes generated by the second generation of both groups of dendrimers with MTX and 5-FU caused a decrease in the mitochondrial potential of 50% for both cell lines. A similar effect was observed for DOX nanocomplexes. The values of mitochondrial potential decreased 40% and 30% vs. control for MCF7 cells and HEPG2, respectively.

### 2.5. Confocal Microscopy Imaging and Flow Cytometry Analysis of Cell Cycle and Apoptosis

**Figure 6:**
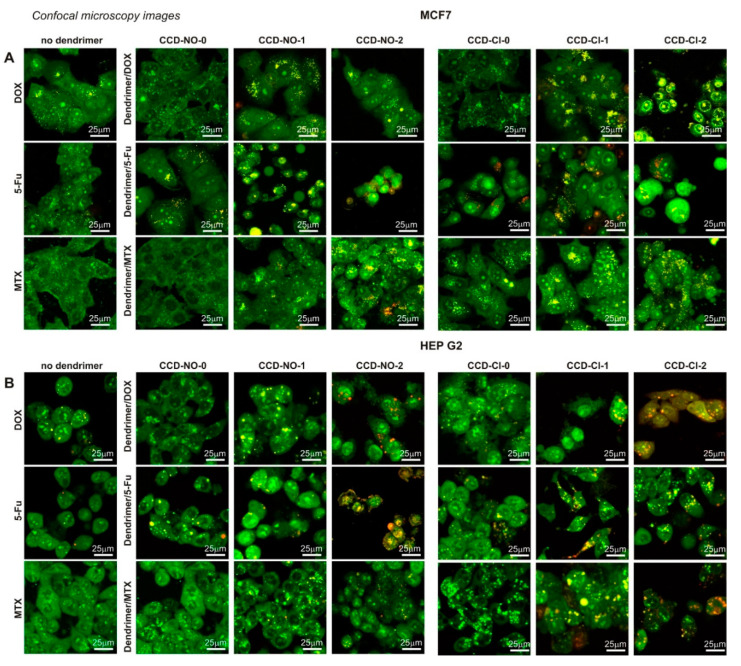
Images of MCF7 (**A**) and HEP G2 (**B**) cells after 48 h exposure to dendrimers, anticancer drugs or dendrimer drug complexes. Incubation time 48 h. The concentrations of drugs were as follows: DOX, 0.1 µmol/L (DOX/dendrimer molar ratio 1:7); 5-FU, 1 µmol/L (5-FU/dendrimer molar ratio 1:1); MTX, 2 nmol/L (MTX/dendrimer molar ratio: 1:32). Cells were stained with orange acridine (OA) and ethidium bromide (EB) and visualized by confocal microscopy. Living cells: morphologically normal (green nucleus); early apoptotic cells: condensed or fragmented chromatin (green nucleus); late apoptotic cells: fragmented and condensed (red chromatin); necrotic: red morphologically normal cells. Scale bar = 25 µm.

The results obtained previously in cell viability, mitochondrial function and ROS level have been connected with the induction of apoptosis and necrosis involved by the studied formulations in MCF7 and HEP G2 cells. Obtained confocal images show visible morphological changes in cells incubated with drugs and dendrimer/drug complexes. Since naked drugs in applied concentrations were not toxic, they were not considered in imaging experiments. Results show that 48 h incubation of cells with dendrimer/drug complexes led to a decrease in the fraction of healthy cells in both cell lines, while the early apoptotic and necrotic cell fractions were significantly increased compared to the cells treated by noncomplexed drugs (Figure 6). These results have good correlation with cytotoxicity studies.

**Figure 7:**
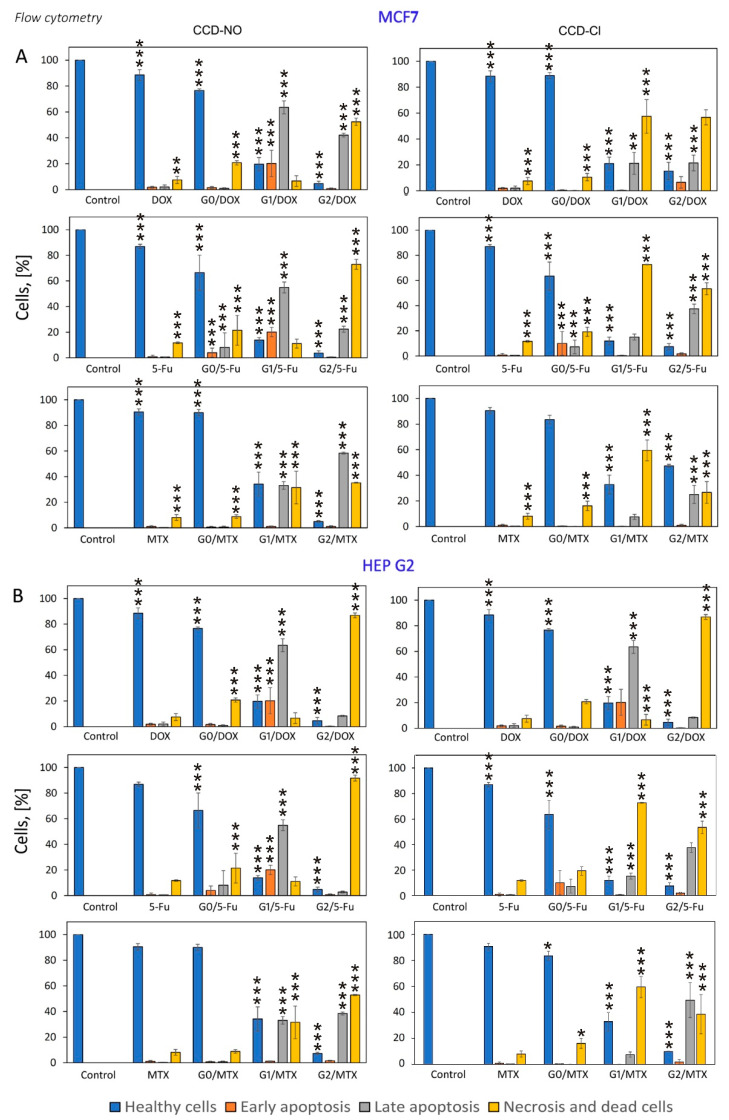
The percentages of cells in different phases and apoptosis profile evaluated by flow cytometry and measured using annexin V/propidium iodide staining in PBS 10 mmol/L, pH 7.4. MCF7 (**A**) and HEP G2 (**B**) cells interacted with dendrimers, anticancer drugs or dendrimer drug complexes. Incubation time 48 h. The concentrations of drugs were as follows: DOX, 0.1 µmol/L (DOX/dendrimer molar ratio 1:7); 5-FU, 1 µmol/L (5-FU/dendrimer molar ratio 1:1); MTX, 2 nmol/L (MTX/dendrimer molar ratio: 1:32). Results are means ± SD, from a min. 3 independent experiments. Statistically significant differences vs. control: * *p* < 0.05, ** *p* < 0.01, *** *p* < 0.001.

To further identify the apoptotic and necrotic fractions formed in MCF7 and HEP G2 cells in the presence of nanocomplexes, the flow cytometry analysis of double-stained annexin V/PI was applied. The 24 h incubation of cells for all studied formulations did not cause significant changes in the analyzed cell fractions (Figure S2). Increasing the incubation time to 48 h shows that the highest percentage of the healthy cell fraction was accounted for after their treatment with noncomplexed drugs (Figure 7). This trend was observed for both studied cell lines. Results indicate that similar low changes in cell fractions were visible when cells were incubated with nanocomplexes formed with compounds of generation “0” (CCD-Cl-0 and CCD-NO-0). However, there was a significant decrease in the percentage of the healthy cell fraction when complexes containing the dendrimers of generations one and two were applied. In that case, the percentage of late apoptotic and necrotic cell fractions increased significantly. The highest percentage increase, >60% of a late apoptotic fraction, was registered for the CCD-Cl-1/5-FU nanocomplex incubated with HEP G2 cells, whereas the biggest amount of necrotic and dead cells (>90%) was caused by the presence of CCD-Cl-2/DOX and CCD-Cl-2/5-FU nanocomplexes (Figure 7).

## 3. Discussion

Cancer treatment is still struggling with many difficulties, one being the side effects of classical chemotherapy. Therefore, numerous studies are focused on new therapeutic approaches such as combinatory therapy or the use of drug carriers that would increase the bioavailability, biocompatibility and stability of conventional drugs and ensure their selective delivery to cancer cells. Due to unique characteristics, dendrimers seem to be good candidates for therapeutic agents or as platforms for delivering chemotherapeutic agents directly to neoplastic cells [6,33,34]. Here, we have evaluated the effect of the combination of carbosilane metallodendrimers with peripheral copper atoms, which have shown anticancer activity in several tumor lines together with conventional drugs such as DOX, MTX and 5-FU that are currently used in clinics. We focused on the evaluation and biophysical characteristics of nanosystems formed by copper (II) carbosilane dendrimers and anticancer drugs. We have also carried out several studies to elucidate the mechanism of cell death when cells are treated with the conjugates.

As expected, the measurements of the zeta potential and size of dendrimer/drug complexes showed increased values with rising dendrimer concentrations in all tested formulations, and the zeta potential and size were lower when complexes were formed with compounds of G0 regardless of metal coordination spheres containing either chloride or nitrate (CCD-NO and CCD-Cl). However, the dendriplexes containing 5-FU showed a sharp drop in their hydrodynamic diameter. The increased surface charge of formed complexes could improve their interaction with cell membranes facilitating the delivery of complexes into cells [34,35,36]. The same tendency was observed previously in a study of the interaction of carbosilane ruthenium dendrimers (CRD) of the first and second generations with MTX, DOX and 5-FU [16].

As previously mentioned, a therapeutic approach in the treatment of cancer is the co-administration of drugs to obtain a synergistic effect in therapeutic activity using lower doses of both drugs, implying a reduction of side effects. To determine the existence of a synergistic effect between the copper dendrimers and the drugs selected for this work, MCF7 and HepG2 cell lines were chosen.

The anticancer activity exhibited by copper (II) metallodendrimers against leukemic 1301, HL-60 and U937 cancer cells had a slight effect on the viability of normal PBMC cells [6,24]. Therefore, our first step was to measure the subtoxic concentrations of drugs and dendrimers against MCF7 and HepG2 in order to determine the concentration of drugs that reduce the cell viability to approximately 80%.

All three generations of compounds were shown to be non-toxic in both cell lines at concentrations <1 µM with different nanoconjugates prepared by keeping a constant drug concentration and increasing the dendrimer concentration to safe concentration levels.

The cytotoxicity studies presented in this work show that 72 h of incubation of MCF7 and HepG2 cells with dendrimer/drug complexes significantly reduced the number of living cells compared to the effect of noncomplexed drugs or dendrimers.

The cytotoxicity of G0/drugs complexes were lower than the effect of formulations prepared with a higher generation of dendrimers (G1/drugs and G2/drugs), probably due to the interaction strength between different drugs and dendritic systems. Interactions are weaker with smaller systems such as zero-generation compounds, with the reduced ability of these dendrimers to cross the membrane barrier and transfect the cells.

On the other hand, the smaller number of metal centers in the zero-generation system compared to the first- and second-generation dendrimers makes them less cytotoxic. Again, these results are in agreement with similar effects observed previously for ruthenium dendrimers complexed with DOX, 5-FU and MTX against HL-60 and 1301 cells [16].

The analysis of the different data obtained indicated that in general and for both cell lines, it is possible to observe that combinations with first-generation metallodendrimers with chloride (CCD-Cl-1) or nitrate ligands (CCD-NO-1) exhibit matching activities. However, a slight increase in activity is observed for second-generation formulations with CCD-NO-2 and a significant decrease for CCD-Cl-2, despite having a higher number of metal centers on the surface. This is in agreement with previous EPR studies carried out for this type of system showing that the dendritic generation and metal center counterion have a clear influence in the interaction with tumor cells and subsequently the effectivity and selectivity of the therapy [24]. In the case of MCF7 cells, the combination of dendritic structures and drugs showed the greatest synergistic effect with methotrexate by decreasing viability around 95.25% vs. control. Similar to previous results, cytotoxicity increases with increasing concentration of the dendritic system in the nanoconjugates. It has been previously demonstrated that the PAMAM dendrimer conjugated with MTX induced a synergistic effect towards MES-SA endometrial cancer cells [8]. Moreover, a third-generation copper (II) metallophosphate dendrimer in combination with DOX, cisplatin, paclitaxel and MG132 showed stronger inhibition of HL60 cell proliferation compared to drugs and dendrimer used alone. However, no synergistic properties were found for the combinations of this dendrimer with camptothecin [37].

It is well known that cytotoxicity effects can be tightly connected with intracellular reactive oxygen species generation [38]. Considering the important role of ROS production for cell viability and the possibility of using this parameter as an indicator of apoptosis regulation [39], we studied the changes of intracellular ROS level with the presence of dendrimer/drug complexes. Despite one of the mechanisms associated with the antiproliferative effects of copper metallodendrimers being ROS production, our current studies showed that at subtoxic concentrations, copper metallodendrimers did not generate an increase of ROS. However, when they were combined with the different drugs tested it was possible to observe the significantly increased level of ROS compared to noncomplexed drugs or noncomplexed dendrimers. The highest increase of ROS levels reached 160% vs. control, in both MCF7 and HepG2 cells, and was observed after 24 h of incubation in selected cases. Following incubation (48 h), this parameter dropped below control values.

Increased and uncontrolled generation of ROS can lead to a situation where the ion channels in the mitochondrial membrane are opened [40]. The open channels result in a collapse of the mitochondrial potential and additional increased production of ROS. The consequence of these changes occurring in the mitochondria can be cell death [41,42,43]. Therefore, we analyzed the changes of mitochondrial potential (ΔΨ_m_) in the presence of the studied nanocomplexes and compared these results with the data obtained for ROS level. While the incubation of cells with naked drugs or dendrimers did not influence this parameter at the tested concentrations, the presence of drug/dendrimer nanosystems significantly depolarized the mitochondrial membrane. These results have good correlation with those obtained for ROS experiments, indicating that this misbalance in the redox status was present in the alteration of mitochondrial membrane potential, again with higher activity of complexes containing dendrimers of generations one and two.

It is known that mitochondrial depolarization is characteristic of the early stages of apoptosis [41,42,44]. Thus, the percentage of apoptotic and necrotic cells in the cell suspension was determined after treatment with dendrimer/drug complexes. According to results obtained by flow cytometry (AV/PI double staining) and confocal microscopy (OA/EB fluorescent staining), the studied nanosystems induced apoptosis and necrosis in both applied cell lines. During 48 h incubation of cells with the dendrimer/drug nanosystems, the highest degree of apoptosis was observed for complexes formed with dendrimers of the first and second generations. For MCF-7 cells, it was mainly early apoptosis, but late apoptosis prevailed in HEP G2 cells. Higher dendrimer generations and concentrations led to the increase of percentage of necrotic and dead cells. In contrast, complexes containing dendrimers of generation “0” were practically not apoptotic in effect, with a high percentage of healthy cells. The results obtained by flow cytometry correlate with data from confocal microscopy. Obtained microimages show that treatment of the cells with dendrimer/drug complexes led to an increase of the number of apoptotic and necrotic MCF7 and HEP G2 cells. Similar results were shown by amphiphilic dendritic nanomicelle. The 12 h incubation of MDA-MB-231 cells with nanocomplex contained dendritic micelles, and DOX or 5-FU initiated the apoptosis and necrosis in cell suspensions [45]. Confocal microscopy images show that the morphology of cells treated by nanocomplexes was influenced strongly when compared to cells incubated with noncomplexed drugs.

## 4. Materials and Methods

### 4.1. Metallodendrimers

**Figure 8:**
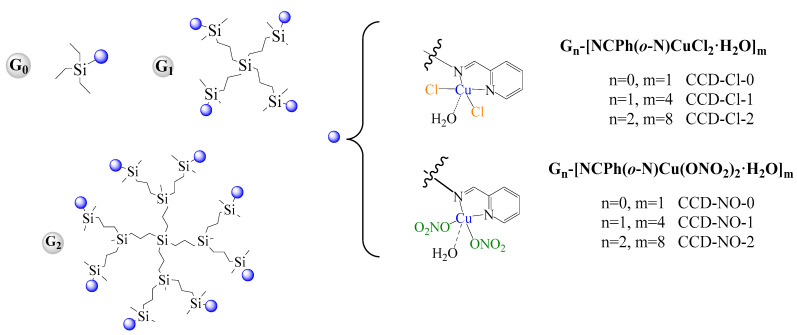
Dendritic structures of Gn-[NCPh(*o*-N)CuCl_2_·H_2_O]m (CCD-Cl) and Gn-[NCPh(o-N)Cu(ONO_2_)_2_·H_2_O]m (CCD-NO) systems.

Two families of copper carbosilane metallodendrimers containing chloride ligands were used: Gn-[NCPh(*o*-N)CuCl_2_·H_2_O]_m_ (n = 0, m = 1 CCD-Cl-0; n = 1, m = 4 CCD-Cl-1; n = 2, m = 8 CCD-Cl-2) and nitrate ligands Gn-[NCPh(*o*-N)Cu(ONO_2_)_2_·H_2_O]m (n = 0, m = 1 CCD-NO-0; n = 1, m = 4 CCD-NO-1; n = 2, m = 8 CCD-NO-2) (structures are shown in Figure 8). The compound synthesis procedure was carefully described in [46]. The basic parameters of dendrimers are given in Table 1.

### 4.2. Drugs

Three commercially available anticancer drugs, doxorubicin (DOX), methotrexate (MTX) and 5-flurouracil (5-FU), were used in the current study for their complexes with copper dendrimers. The drugs were purchased from Sigma-Aldrich Sp. Z.O.O., Poznan, Poland.

### 4.3. Zeta Size

The hydrodynamic diameter of the CCD/drug complexes was estimated by dynamic light scattering technique using the Malvern Zetasizer Nano ZS-90 spectrometer, (Malvern Instruments, Malvern, UK). Complexes were prepared in distilled water. Drug concentration was always 10 µmol/L, and the concentration of dendrimers varied from 1:0.25 to 1:75 of dendrimer/drug molar ratio. For each sample, 15 measurements in 5 cycles were taken at room temperature. The experiment was repeated 3 times for each compound. The data were analyzed by Malvern software and presented as a mean ± standard deviation (SD).

### 4.4. Zeta Potential

Zeta potential values of formed complexes were evaluated by the Laser Doppler Velocimetry technique using the Zetasizer Nano ZS-90, Malvern Instruments UK. The Helmholtz–Smoluchowski equation was applied to calculate zeta potential. Complexes were prepared in distilled water. Drug concentration was always 10 µmol/L, and the concentration of dendrimers varied from 1:0.25 to 1:75 of dendrimer/drug molar ratio. For each sample, 15 measurements in 5 cycles were made at room temperature. The experiment was repeated 3 times for each compound. The data were analyzed by Malvern software and presented as a mean ± standard deviation (SD).

### 4.5. Cells

To determine the effect of studied dendrimer/drug complexes on the cell viability level of reactive oxygen species, mitochondrial membrane potential and the percentage of apoptotic and necrotic cells, 2 cancer cell lines, MCF-7 (human breast cancer cell line) and HepG2 (human liver carcinoma cell line), were used. Cells were purchased from ATCC Company, Manassas, VA, USA. The cells were grown in plastic tissue culture flasks (Falcon, GE Healthcare Life Sciences, Chicago, IL, USA) at the temperature of 37 °C in a humidified atmosphere containing 5%, CO_2_ and 95% air. DMEM (Gibco, Thermo Fisher Scientific, Waltham, MA, USA) with 10% heat-inactivated fetal bovine serum (FBS, HyClone, GE Healthcare Life Sciences, Chicago, IL, USA) containing 1% antibiotic for cell culture was used.

### 4.6. Cytotoxicity

To evaluate the cytotoxicity effects of studied complexes, the MTT (Sigma, USA) assay was applied. Cells were seeded on a 96-well plastic plate at 1 × 10^4^ cells per well and incubated with formed dendriplexes for 72 h. Next, the 3-(4,5-dimethylthiazol-2-yl)-2,5-diphenyltetrazolium bromide (MTT) solution was added. To dissolve the formazan crystals, the DMSO was added after 3 h of incubation. Absorbance of samples was measured at 580 nm and 720 nm. The cells’ viability was calculated using following equation:

% viability = A/A_c_ × 100

where A is the absorbance measured for sample; A_c_ is the absorbance of the control cells. The results are presented as mean ± standard deviation (SD) from 3 independent experiments.

### 4.7. ROS Level

The H_2_DCFDA (Thermo Fisher Scientific, Waltham, MA, USA) fluorescent probe was used to measure the changes of the reactive oxygen species level in the cells treated by dendrimer/drug complexes. H_2_DCFDA is not able to emit fluorescence until the acetate groups are removed by esterases inside of the cell. During the oxidation process, highly fluorescent DCF (dichlorofluorescein) formed from H_2_DCF. The changes in fluorescence intensity of DCF reflect the alterations in the intracellular level of ROS. Cells were seeded on a 96-well black plate, at 1.5 × 10^4^ cells per well. Cells were incubated with dendriplexes for 0.5, 3, 24 and 48 h, the medium was removed, and cells were washed with PBS. Next, the H_2_DCFDA probe at final concentration 2 µmol/L was added in each well. Cells were incubated for 15 min at 37 °C and washed with PBS. After these procedures, the fluorescence was measured at the excitation and emission wavelength of 485 nm and 530 nm, respectively. The results are presented as mean ± standard deviation (SD) from 3 independent experiments.

### 4.8. Mitochondrial Membrane Potential

The fluorescent probe JC-1 (5,5′,6,6′-tetrachloro-1,1′,3,3′-tetraethylbenzimidazolylcarbocyanine iodide) (Thermo Fisher Scientific, Waltham, MA, USA) was used to determine changes in mitochondrial membrane potential (Ψ_m_) for cells treated with dendrimer/drug complexes. JC-1 accumulates in mitochondria and exhibits red fluorescence with an excitation and emission wavelength of 530 and 590 nm, respectively. When the mitochondrial membrane is depolarized, JC-1 exhibits green fluorescence with an excitation and emission wavelength of 485 and 540 nm, respectively. An increased ratio in the red-to-green fluorescence can indicate the increase of Ψ_m_. As in the previous technique, the cells were seeded on a 96-well black plate at 1.5 × 10^4^ cells per well. After 24 and 48 h of incubation of cells with dendriplexes, the medium was removed, and cells were carefully washed with PBS. After that, cells were incubated for 20 min at 37 °C, in the presence of JC-1 at the final concentration of 5 µmol/L. Cells were then washed with PBS. Measurements were made at an excitation and emission wavelength of 530 nm and 590 nm, respectively. The results are presented as mean ± standard deviation (SD) from 3 independent experiments.

### 4.9. Annexin V/Propidium Iodide Double-Staining Assay

Flow cytometry technique using annexin V-FITC Apoptosis Detection Kit was applied to determine the percentage of apoptotic and necrotic cells after their treatment with dendrimer/drug complexes. The final concentrations of drugs were as follows: DOX, 0.1 µmol/L (DOX/dendrimer molar ratio 1:7); 5-FU, 1 µmol/L (5-FU/dendrimer molar ratio 1:1); MTX, 2 nmol/L (MTX/dendrimer molar ratio: 1:32). Cells were incubated with drugs or dendrimer/drug complexes for 24 and 48 h and washed twice in cold PBS. After washing, cells were suspended with a binding buffer (HEPES/NaOH, pH 7.4). The cell suspension was adjusted for a concentration of 10^6^ cells/mL. An amount of 100 µL of the suspension was then placed in a measuring tube, and 5 µL of annexin V-FITC + 10 µL of propidium iodide (PI) was added and mixed.

The following controls samples were additionally prepared: (1) negative control (unlabeled cells), (2) PI positive control (cold ethanol was used to induce necrosis), (3) annexin V positive control (hydrogen peroxide was used to induce apoptosis). Each sample was analyzed by flow cytometry (LSRII, Becton Dickinson, NJ, USA). The results are presented as mean ± standard deviation (SD) from 3 independent experiments.

### 4.10. Orange Acridine/Ethidium Bromide Fluorescent Staining

The ability of dendrimer/drug complexes to induce the apoptosis and necrosis in MCF7 and HEP G2 cells was evaluated by confocal microscopy using double fluorescent dye staining of orange acridine (OA) and ethidium bromide (EB). Both of these fluorescent dyes can stain cell nuclei after intercalation with DNA. While OA can be easily uptaken by cells and stains the nucleus green, EB is able to stain the nucleus of damaged cells only in red. Confocal analysis allows the indication of the fractions of the cells that are early and late apoptotic, necrotic or healthy. The cells were treated with dendriplexes for 24 h and stained with dual fluorescent staining solution (2 μL) containing 100 μg/mL AO and 100 μg/mL EB for 2 min and covered with a coverslip. Then, cells were washed with PBS and visualized using a Leica TCS SP8 confocal microscope (Wetzlar, Germany) with the objective 63×/1.40 (HC PL APO CS2, Leica Microsystems, Wetzlar, Germany).

The cells with normal morphology and a green nucleus were recognized as living cells, with green nucleus and condensed or fragmented chromatin as early apoptotic cells, with condensed or fragmented red chromatin as late apoptotic cells and with red nucleus as necrotic cells. Leica Application Suite X (LAS X, Leica Microsystems, Germany) software was used for the export of images.

### 4.11. Statistical Analysis

The results come from a minimum of 3 independent experiments. To assess the significance of differences, one-way analysis of variance (ANOVA) and Bonferroni test were applied. Significance was accepted at *p* ≤ 0.05 or less.

## 5. Conclusions

In summary, conjugation of copper carbosilane metallodendrimers with doxorubicin, 5-florouracyl or methotrexate at subtoxic concentrations appears to be a promising strategy that could be considered for anticancer drug delivery, due to the synergistic effect of the used components. In vitro experiments show that copper dendrimers significantly increase the anticancer effect of the applied drugs and probably can serve as drug transporters. The synergistic effect of dendrimer/drug complexes has been confirmed by several biophysical assays. Rapid increase of the ROS level and depolarization of mitochondrial membranes caused by the action of nanocomplexes indicated their ability to induce apoptosis in MCF7 and HEP G2 cancer cells. Observed cytotoxicity associated with the onset of cell apoptosis and necrosis depended on the generation of dendrimers. In vivo experiments are planned to determine the ability of the studied systems for cancer therapy.

## Figures and Tables

**Table 1:** Characterization of copper metallodendrimers with chloride and nitrate ligands.

Compound	Generation/Number of Surface Groups	Molecular Weight (g/mol)
CCD-Cl-0	0/1	414.93
CCD-Cl-1	1/4	1627.68
CCD-Cl-2	2/8	3696.01
CCD-NO-0	0/1	468.04
CCD-NO-1	1/4	1840.10
CCD-NO-2	2/8	3992.90

## Data Availability

The data presented in this study are available on request from the corresponding author.

## Supplementary Materials

The following supporting information can be downloaded at: https://www.mdpi.com/article/10.3390/ijms24044076/s1.
