# Supplementary material for: Combination of Copper Metallodendrimers with Conventional Antitumor Drugs to Combat Cancer in In Vitro Models
Source: Int J Mol Sci. 2023 Feb 17;24(4):4076. doi: 10.3390/ijms24044076 (PMC9960994; doi:10.3390/ijms24044076)
Supplement: Supplementary file 1 [file ijms-24-04076-s001.zip › ijms-2206058-supplementary.pdf]

Supporting information

Combination of copper metallodendrimers with conventional antitumor drugs to combat cancer in *in vitro* models

Marcin Hołota [1], Sylwia Michlewska [1,2*], Sandra Garcia-Gallego [3,4,5], Natalia Sanz del Olmo [3], Paula Ortega [3,4,5], Maria Bryszewska[1], Francisco Javier de la Mata [3,4,5], Maksim Ionov [1]

Department of General Biophysics, Faculty of Biology & Environmental Protection, University of Lodz, Pomorska 141/143, 90–236 Lodz, Poland
[2] Laboratory of Microscopic Imaging & Specialized Biological Techniques, Faculty of Biology & Environmental Protection, University of Lodz, Banacha12/16, 90–237 Lodz, Poland
[3] Department of Organic and Inorganic Chemistry, Research Institute in Chemistry “Andrés M. del Río” (IQAR), Universidad de Alcalá, 28805 Madrid, Spain
[4] Networking Research Center on Bioengineering, Biomaterials and Nanomedicine (CIBER-BBN), 28029 Madrid, Spain
[5] Institute Ramón y Cajal for Health Research (IRYCIS), 28034 Madrid, Spain
* Correspondence: sylwia.michlewska@biol.uni.lodz.pl

The copper metallodendrimers with chloride and nitrate ligands were synthesized accordingly with previously published protocol (*Sanz Del Olmo, N., Maroto-Díaz, M., Gómez, R., Ortega, P., Cangiotti, M., Ottaviani, M. F., & de la Mata, F. J. (2017). Carbosilane metallodendrimers based on copper (II) complexes: Synthesis, EPR characterization and anticancer activity. Journal of inorganic biochemistry, 177, 211–218.* https://doi.org/10.1016/j.jinorgbio.2017.09.023). Its purity was evaluated by elemental analysis, nuclear magnetic resonance and mass spectrometry techniques.

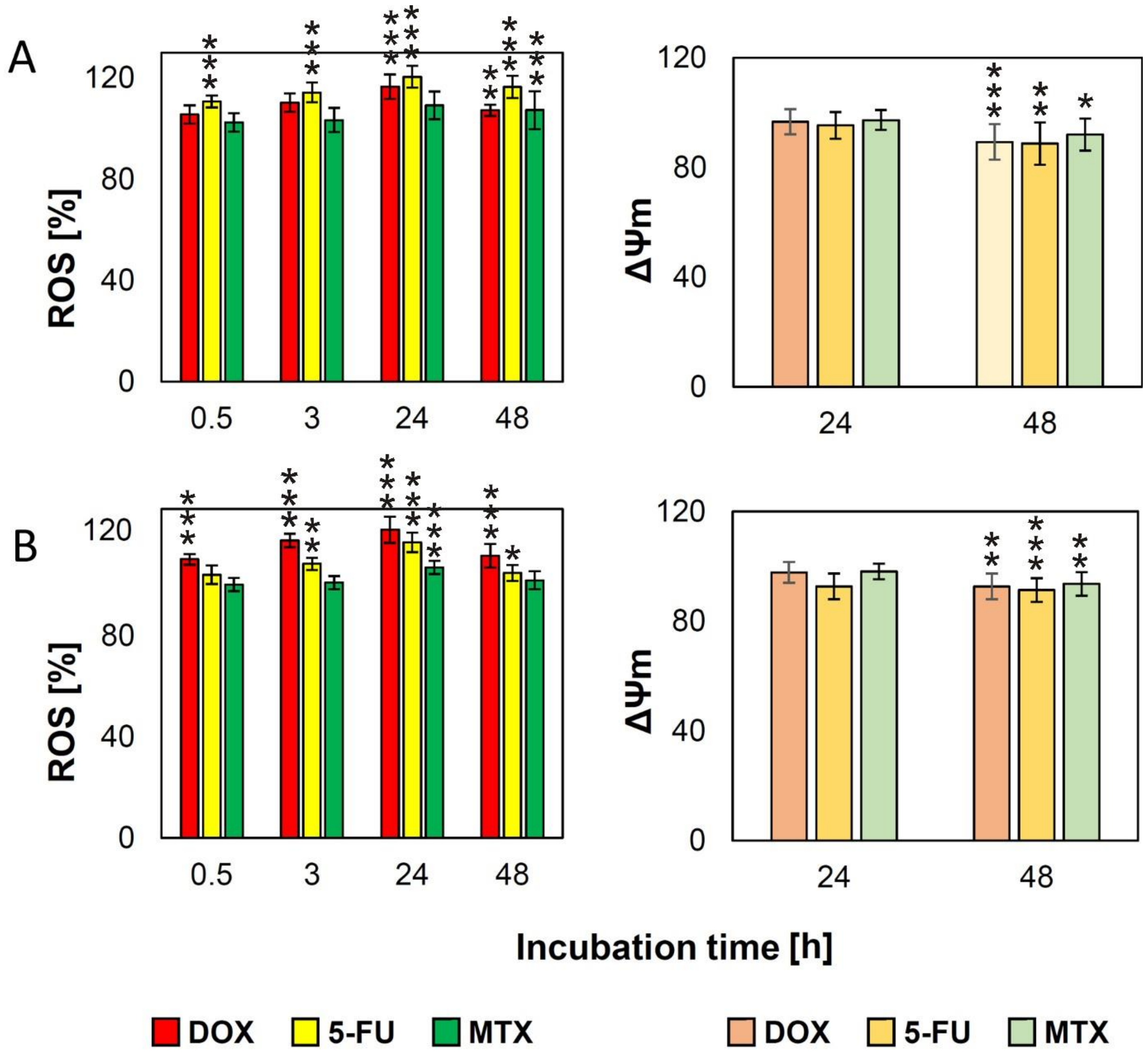

Figure S1. Time-dependent ROS production and changes in mitochondrial membrane potential (Ψm**)** in MCF7 – (A) or HEP G2 – (B) cells involved by anticancer drugs DOX, 5-Fu and MTX. ROS - fluorescent probe H_2DCFDA; ΔΨm - fluorescent probe 5,5′,6,6′-tetrachloro-1,1′,3,3′-tetraethylbenzimidazolylcarbocyanine iodide (JC-1), PBS 10 mmol/L, pH-7.4. Results are means ± SD, from a min. of 3 independent experiments. Statistically significant differences vs. control *p <0.05, **p < 0.01, ***p < 0.001.

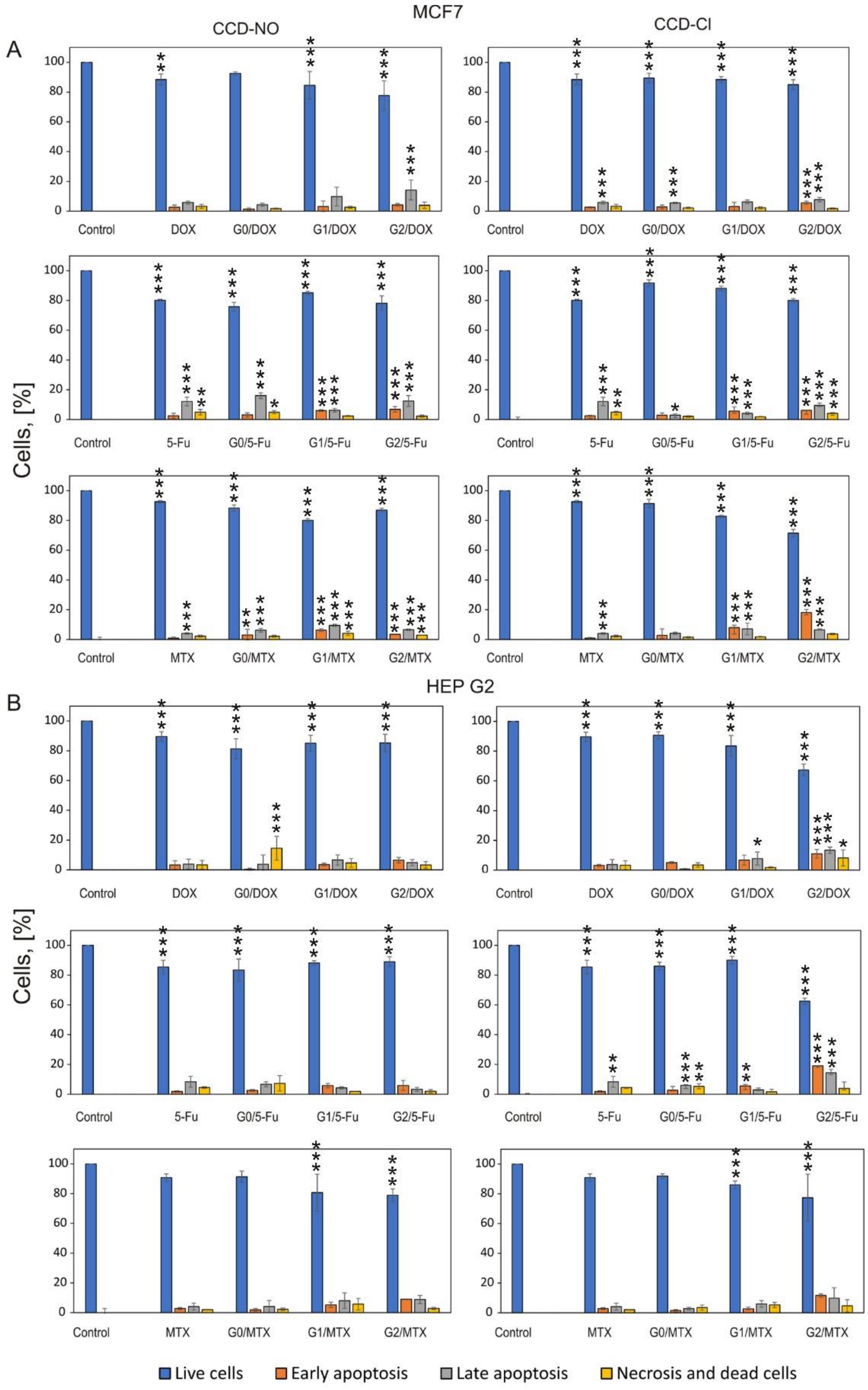

Figure S2. The percentages of cells in different phases and apoptosis profile evaluated by flow cytometry and measured using annexin V/propidium iodide staining in PBS 10 mmol/L, pH-7.4. MCF7 – (A) and HEP G2 – (B) cells were interacted with dendrimers, anticancer drugs or dendrimer drug complexes. Incubation time 24 h. The concentrations of drugs were as follows: DOX, 0.1 µmol/L (DOX/Dendrimer molar ratio 1:7); 5-FU, 1 µmol/L (5-FU/Dendrimer molar ratio 1:1); MTX, 2 nmol/L (MTX/Dendrimer molar ratio:

1:32). Results are means ± SD, from a min. of 3 independent experiments. Statistically significant differences vs. control *p <0.05, **p < 0.01, ***p < 0.001.
